# Development of targeted, theory-informed interventions to improve bronchiolitis management

**DOI:** 10.1186/s12913-021-06724-6

**Published:** 2021-08-03

**Authors:** Libby Haskell, Emma J. Tavender, Catherine L. Wilson, Sharon O’Brien, Franz E. Babl, Meredith L. Borland, Elizabeth Cotterell, Nicolette Sheridan, Ed Oakley, Stuart R. Dalziel, on behalf of the Paediatric Research in Emergency Departments International Collaborative (PREDICT) network, Australasia

**Affiliations:** 1 Children’s Emergency Department, Starship Children’s Hospital, Private Bag 92024, Auckland, 1142 New Zealand; 2 Department of Paediatrics: Child and Youth Health, University of Auckland, Auckland, New Zealand; 3 Clinical Sciences, Murdoch Children’s Research Institute, Parkville, Melbourne, Victoria Australia; 4 Departments of Paediatrics and Critical Care, University of Melbourne, Melbourne, Victoria Australia; 5 Emergency Department, Perth Children’s Hospital, Perth, Western Australia Australia; 6 Curtin University, Perth, Western Australia Australia; 7 Emergency Department, The Royal Children’s Hospital, Melbourne, Victoria Australia; 8 Divisions of Emergency Medicine and Paediatrics, School of Medicine, University of Western Austalia, Western Australia, Australia; 9 Armidale Rural Referral Hospital, Armidale, New South Wales Australia; 10 School of Rural Medicine, University of New England, Armidale, New South Wales Australia; 11 College of Health, Massey University, Auckland, New Zealand; 12 Department of Surgery, University of Auckland, Auckland, New Zealand

**Keywords:** Intervention, De-implementation, Theoretical domains framework, Behaviour change techniques, Bronchiolitis

## Abstract

**Background:**

Despite international guidelines providing evidence-based recommendations on appropriate management of infants with bronchiolitis, wide variation in practice occurs. This results in infants receiving care of no benefit, with associated cost and is potentially harmful. Theoretical frameworks are increasingly used to develop interventions, utilising behaviour change techniques specifically chosen to target factors contributing to practice variation, with de-implementation often viewed as harder than implementing. This paper describes the stepped process using the Theoretical Domains Framework (TDF) to develop targeted, theory-informed interventions which subsequently successfully improved management of infants with bronchiolitis by de-implementing ineffective therapies. Explicit description of the process and rationale used in developing de-implementation interventions is critical to dissemination of these practices into real world clinical practice.

**Methods:**

A stepped approach was used: (1) Identify evidence-based recommendations and practice variation as targets for change, (2) Identify factors influencing practice change (barriers and enablers) to be addressed, and (3) Identification and development of interventions (behaviour change techniques and methods of delivery) addressing influencing factors, considering evidence of effectiveness, feasibility, local relevance and acceptability. The mode of delivery for the intervention components was informed by evidence from implementation science systematic reviews, and setting specific feasibility and practicality.

**Results:**

Five robust evidence-based management recommendations, targeting the main variation in bronchiolitis management were identified: namely, no use of chest x-ray, salbutamol, glucocorticoids, antibiotics, and adrenaline. Interventions developed to target recommendations addressed seven TDF domains (identified following qualitative clinician interviews (*n* = 20)) with 23 behaviour change techniques chosen to address these domains. Final interventions included: (1) Local stakeholder meetings, (2) Identification of medical and nursing clinical leads, (3) Train-the-trainer workshop for all clinical leads, (4) Local educational materials for delivery by clinical leads, (5) Provision of tools and materials targeting influencing factors, and prompting recommended behaviours, and (6) Audit and feedback.

**Conclusion:**

A stepped approach based on theory, evidence and issues of feasibility, local relevance and acceptability, was successfully used to develop interventions to improve management of infants with bronchiolitis. The rationale and content of interventions has been explicitly described allowing others to de-implement unnecessary bronchiolitis management, thereby improving care.

**Supplementary Information:**

The online version contains supplementary material available at 10.1186/s12913-021-06724-6.

## Background

Changing clinicians’ practice is challenging, in part due to the difficulty of improving quality and safety in healthcare [1] and exacerbated by inappropriate methods used to design interventions aiming to improve practice, with lack of explicit rationale for the intervention choices made [2]. Developing interventions is complex and the use of theory in the intervention development process is recommended [3], with interventions being more likely to be effective if targeting causal determinants of behaviour and behaviour change [4]. Better description and justification of interventions chosen has been recommended to enable replication and refinement of interventions [5–7]. The Theoretical Domains Framework (TDF) was designed to incorporate a wide range of behaviour change theories for use in implementation research with subsequent validation [8, 9]. The TDF has demonstrated strong explanatory and predictive powers across a number of healthcare settings, including acute care settings, and is particularly useful when selecting interventions to improve practice [10, 11]. A key benefit of using the TDF is that behaviour change techniques (BCTs) are linked to each TDF domain, enabling utilisation of BCTs most likely to tackle issues identified [12], with guidance available to assist in achieving implementation objectives [13].

Bronchiolitis is the most common cause for hospitalisation of infants less than 1 year of age. Management is well defined [14] with all international evidence-based guidelines consistently recommending supportive care; and against the use of chest x-ray (CXR), salbutamol, antibiotics, glucocorticoids, or adrenaline [15–18]. Despite these consistent recommendations, and campaigns such as Choosing Wisely which aims to promote a culture of avoiding inappropriate treatments, [19] significant variation in management of infant’s with bronchiolitis remains with infants often receiving management of no benefit, and potential risk of harm [20, 21]. It is for these reasons that bronchiolitis was chosen as an appropriate condition for a de-implementation trial [22, 23].

This paper details the development of targeted, theory-informed interventions to address influencing factors identified previously [24], with the explicit aim to improve management of infants with bronchiolitis in both the emergency department (ED) and paediatric inpatient units by de-implementing the use of therapies known to be of no benefit. Subsequent to the development of these interventions they have been robustly assessed in a multi-centre cluster randomised controlled trial (cRCT) involving 26 hospitals. Results from our trial demonstrated a 14.1% risk difference favouring the intervention group in compliance to five key bronchiolitis guideline recommendations, measurably improving the management of infants with bronchiolitis [25, 26].

## Methods

**Fig. 1:**
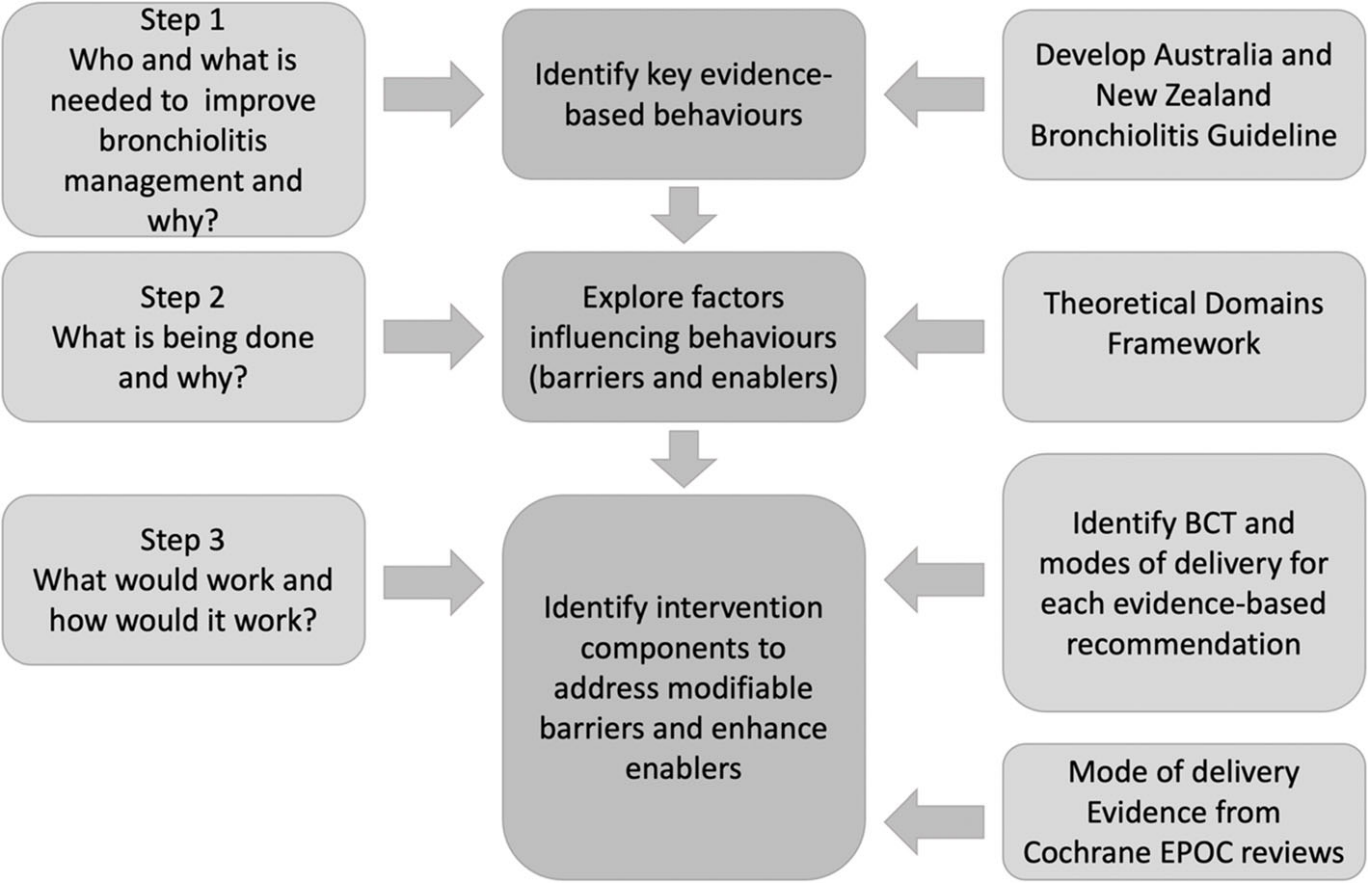
Process of developing targeted, theory-informed interventions^1^. BCT – Behaviour Change Technique. EPOC – Effective Practice and Organisation of Care. ^1^Adapted from French et al. [27] and Tavender et al. [28]

We used a stepped approach to develop targeted, theory-informed bronchiolitis interventions (Fig. 1). This logical approach for developing complex interventions is based on theory, evidence and practical issues [27], and has been successful in acute care settings [28, 29]. A three-stepped method was undertaken:

Step 1: Who and what is needed to improve bronchiolitis management?

Step 2: Using a theoretical framework, which barriers and enablers need to be addressed?

Step 3: Which intervention components (BCTs and mode(s) of delivery) could overcome the modifiable barriers and enhance the enablers?

### Step 1: who and what is needed to improve bronchiolitis management?

#### Identify or develop locally applicable, actionable evidence-based recommendations

In 2015 the Paediatric Research in Emergency Departments International Collaborative (PREDICT) [30] developed the first evidenced-based guideline for the management of bronchiolitis for use in Australia and New Zealand; the Australasian Bronchiolitis Guideline [18]. The guideline aimed to provide clear guidance to clinicians treating infants presenting to EDs and paediatric inpatient units with bronchiolitis. Key evidence-based recommendations from the Australasian Bronchiolitis Guideline were identified based on the strength of the recommendation and supporting evidence.

#### Identify the evidence-practice gap

The literature was searched to review current data on adherence with evidence-based bronchiolitis guideline recommendations from which to target improvement efforts.

### Step 2: using a theoretical framework, which barriers and enablers need to be addressed?

Semi-structured qualitative interviews were conducted with 20 ED and inpatient paediatric unit nurses and doctors. Interview questions used the TDF domains to explore barriers and enablers to practice variation and factors that may influence the uptake of evidence-based bronchiolitis recommendations [25]. For example, ‘Are you confident in assessing an infant with bronchiolitis without doing a CXR [Knowledge]?’ ‘Do you feel that giving salbutamol to infants with bronchiolitis improves outcomes [Beliefs about consequences]?’ Purposeful sampling was used to select a range of clinicians from senior to junior, from metropolitan and regional, and from Australia and New Zealand to interview. Participants completed written informed consent and gave verbal confirmation at the start of the interview. Interview transcripts were coded using thematic content analysis in order to identify TDF domains to target in interventions.

### Step 3: which intervention components (BCTs and mode(s) of delivery) could overcome the modifiable barriers and enhance the enablers?

#### Identify potential BCTs and modes of delivery for each evidence-based recommendation

To select BCTs most likely to effect change for each of the key evidence-based guideline recommendations, we used the BCT matrix validated by Cane et al [9]. This matrix provides guidance on selecting BCTs most likely to address each TDF domain. By matching the key TDF domains identified during our qualitative clinician interviews with BCTs most likely to influence these domains, we expected to increase the likelihood of influencing enablers and barriers to evidence-based bronchiolitis management. Where there were no BCTs assigned based on the Cane et al. matrix [12], additional BCTs identified by Michie et al. were selected [4].

An implementation development panel of eight members including clinicians (senior nurses and doctors from ED and paediatric inpatient units with direct responsibility for the management of infants with bronchiolitis) and an implementation scientist, reviewed the identified TDF domains and related BCTs, with feasible methods of implementation delivery discussed.

#### Identify evidence from systematic reviews of effects of interventions to inform the selection of intervention components

The Cochrane Effective Practice and Organisation of Care (EPOC) group have published systematic reviews of interventions to improve both healthcare systems and healthcare delivery [31–36]. Additional Table 1 details the key findings from the reviews and considerations of implementing them for bronchiolitis management in our clinical setting. These reviews and findings from Grimshaw et al.*’s* summary of interventions [37] were discussed by the implementation development panel and research group to aid selection of appropriate interventions.

#### Identify feasibility, local relevance and acceptability of the intervention

The implementation development panel and research group considered factors to maximise the likelihood that the interventions were feasible, relevant and acceptable in the acute care environment to which they were to be implemented e.g. consideration of education sessions of appropriate length for an acute care environment; clear and succinct feedback reports of regular audits.

Recommendations from the Workgroup for Intervention Development and Evaluation Research (WIDER) [7], the Template for Intervention Description and Replication (TIDieR checklist) [6], and *Proctor* et al. [5] were used to guide describing the intervention components to ensure transparency and replicability. The following criteria were used to operationalise the intervention components: (1) Characteristics of those delivering the intervention, (2) Characteristics of the recipients, (3) The setting, (4) Intervention content, (5) Mode of delivery, (6) Intensity or dose, (7) The duration (number of sessions, time), and (8) Justification (rationale for intervention).

## Results

### Step 1: who and what is needed to improve bronchiolitis management?

#### Identify or develop locally applicable, actionable evidence-based recommendations

The Australasian Bronchiolitis Guideline [18] identified 33 recommendations which were broadly consistent with other international bronchiolitis guidelines [15–17]. From these, five evidence-based recommendations were chosen to target (Table 1). These had the highest quality evidence supporting the recommendations, and were thought to be modifiable at a clinician, departmental or hospital level. These recommendations were to not use CXR, salbutamol, glucocorticoids, antibiotics, and adrenaline in the management of infants with bronchiolitis. As these five recommendations are not independent of each other (e.g. an infant with bronchiolitis who has a CXR is more likely to receive antibiotics; salbutamol and glucocorticoids, and adrenaline and glucocorticoids, are often prescribed concurrently), we chose to develop the intervention package as a whole, as aiming to improve one or two of these recommendations at the expense of others would be difficult to justify.

**Table 1:** Five evidence-based recommendations targeted from Australasian Bronchiolitis Guideline

Clinical intervention	GRADE quality of evidence	Guideline recommendation
Salbutamol	Strong	Do not administer salbutamol to infants, less than or equal to 12 months of age, presenting to hospital or hospitalised with bronchiolitis.
Antibiotics	Conditional	Do not use antibiotics to treat infants with bronchiolitis.
Glucocorticoids	Strong	Do not administer systemic or local glucocorticoids to infants presenting to hospital or hospitalised with bronchiolitis.
Adrenaline	Strong	Do not administer adrenaline to infants presenting to hospital or hospitalised with bronchiolitis.
Chest x-ray	Conditional	Routine chest x-ray is not recommended as it does not improve management in infants presenting with simple bronchiolitis and may lead to treatments of no benefit.

*GRADE* Grading of Recommendations, Assessment, Development and Evaluations

#### Identify the evidence-practice gap

The search of the literature identified a large study conducted by Paediatric Emergency Research Networks (PERN) in 38 EDs in Canada, the United States, Australia, New Zealand, the United Kingdom, Ireland, Spain, and Portugal where more than 30% of infants received non-evidence-based supportive care [21]. In Australia and New Zealand, data from over 3400 bronchiolitis presentations from seven tertiary paediatric hospital providers demonstrated that at least one of the five interventions known to have no benefit was used in 27 to 48% of bronchiolitis admissions, with salbutamol being most likely to be used [20]. These studies provided robust evidence of both an evidence-practice gap and significant variation in practice.

### Step 2: using a theoretical framework, which barriers and enablers need to be addressed?

Interviews with 20 clinicians (12 doctors, 8 nurses) from four Australian and New Zealand hospitals were conducted between July and October 2016. The detailed findings from these interviews have been reported separately [25]. The key barriers for providing evidence-based management for infants with bronchiolitis were associated with seven of the 14 TDF domains. These were beliefs about consequences, knowledge, social/professional role and identity, environmental context and resources, skills, social influences, and belief about capabilities. The first five domains listed were identified as consistently important in four of our five target recommendations. Beliefs about consequences were most notably important in relation to the use of CXR. Clinician’s fear of missing a more serious diagnosis, such as pneumonia, drives the use of CXR with the unwanted consequence of increased antibiotic use associated with having a CXR. This example highlights both lack of knowledge of how to diagnose bronchiolitis, and of confidence in clinician skill of making a clinical diagnosis. The domains of social influences and beliefs about capabilities featured but less prominently. This included perceived or actual pressure from families to prescribe medications (antibiotics, salbutamol, or glucocorticoids) or undertake a CXR, or from other clinicians to trial salbutamol or undertake a CXR. The barriers and enablers identified for bronchiolitis target behaviours detailed by TDF domains are detailed in Table 2, and by target behaviours in Additional Table 2**.**

**Table 2:** Barriers and enablers identified for bronchiolitis target behaviours by Theoretical Domains Framework

TDF Domain	Barriers and enablers (target behaviour)
Beliefs about consequences	Clinician concern that missing an alternative diagnosis e.g., pneumonia, particularly when infant has significant increased work of breathing (CXR, salbutamol, antibiotics). Undertaking investigation will confirm the diagnosis (CXR). Some clinicians believing there is benefit from a trial of the therapy to prevent admission, with others disagreeing (Salbutamol). Conflicting beliefs of little or no harm, or benefit from therapy (CXR, salbutamol, antibiotics, glucocorticoids). Belief that the new bronchiolitis guideline would benefit evidence-based bronchiolitis management; some senior doctors stating the guideline would not change their practice (CXR, salbutamol, glucocorticoids, antibiotics, adrenaline). Infants from deprived populations may benefit from therapy (Antibiotics). Confidence in de-prescribing/ceasing therapy already commenced (Salbutamol, antibiotics, glucocorticoids).
Knowledge	Lack of experience in caring for infants with bronchiolitis (CXR, salbutamol, antibiotics, glucocorticoids). Lack of knowledge of current bronchiolitis evidence (CXR, salbutamol, antibiotics, glucocorticoids, adrenaline).
Social professional role and identity	Nurses supporting junior doctors in caring for infants with bronchiolitis (CXR, salbutamol, glucocorticoids). Nurses being disregarded when questioning treatments (CXR, salbutamol, antibiotics). Junior doctors lacking confidence in contacting seniors for advice (CXR, salbutamol). Importance of medical and nursing teamwork when managing infants with bronchiolitis (CXR, salbutamol, antibiotics, glucocorticoids).
Environmental context and resources	Reduced senior medical support after hours; time pressures in ED leading to undertaking investigation (CXR). Regional hospitals having significant distance to tertiary care and less paediatric trained/experienced staff with more overseas trained doctors who may practice differently leading to investigations and therapies (CXR, salbutamol, antibiotics, glucocorticoids). Challenges with staff turnover and maintain regular bronchiolitis education (CXR, salbutamol, antibiotics, glucocorticoids). Importance of positive relationships between ED and inpatient paediatric units (CXR, salbutamol, antibiotics, glucocorticoids).
Skills	Lack of confidence in diagnosing and managing bronchiolitis (CXR, salbutamol, antibiotics, glucocorticoids). Importance of nursing involvement in bronchiolitis management (CXR, salbutamol, antibiotics, glucocorticoids). Lack of confidence in discussing supportive bronchiolitis management with families/caregivers (CXR, salbutamol, antibiotics, glucocorticoids).
Social influences	Pressure from parent/caregiver and other clinicians to investigate or prescribe therapy (CXR, salbutamol, antibiotics, glucocorticoids).
Beliefs about capabilities	Importance for families/caregivers to maintain positive relationships with primary care providers when ceasing treatments (Salbutamol, glucocorticoids, antibiotics) Wanting to “do something” or prevent deterioration (CXR, salbutamol, antibiotics, glucocorticoids).

*CXR* Chest X-ray

*TDF* Theoretical Domains Framework

*ED* Emergency Department

### Step 3: which intervention components (BCTs and mode(s) of delivery) could overcome the modifiable barriers and enhance the enablers?

#### Identify potential BCTs and modes of delivery for each evidence-based recommendation

Twenty-three BCTs were selected to target barriers and enablers for the evidence-based management of bronchiolitis from seven TDF domains. The domain of social professional role and identity had no specified BCTs in the Cane et al. [12] matrix, therefore the BCT recommended previously by Michie et al. [4] was utilised. Table 3 details the mapping process for selecting BCTs as intervention components.

**Table 3:** Mapping of important barriers and enablers (grouped by TDF) to behaviour change techniques and intervention components developed

TDF Domain	Selected BCTs as intervention components
Beliefs about consequences	**Persuasive communication; Information regarding behaviour, outcome**^**1**^**:** Clinical leads (nursing and medical) will continually educate to reinforce the benefits of adhering to the 5 bronchiolitis guideline recommendations. **Feedback**^**1**^**:** Provide historical data on hospital and Australasian bronchiolitis compliance, with monthly individual hospital audit data being disseminated to clinicians by clinical leads. **Pros and cons**^**2**^**:** Clinical leads to discuss positive impact of following guideline in reducing therapies known to be of no benefit. **Vicarious reinforcement**^**2**^**:** Clinical leads will use reinforcement messages in education of following guideline recommendations. **Social and environmental consequences**^**2**^**; Salience of consequences**^**2**^**:** Clinical leads will reinforce consequences for infants/families and hospital in following guideline e.g. length of stay, cost, reducing harm.
Knowledge	**Information regarding behaviour, outcome**^**1**^**:** Clinical leads will continually educate to reinforce the benefits of adhering to guideline recommendations and use evidence information sheets to reinforce (salbutamol; CXR and antibiotics). **Antecedents**^**2**^: Clinical leads to educate clinicians on situations or events that predict increased therapy use e.g. time and family pressure, reduced senior support. **Health consequences**^**2**^**:** Clinical leads will reinforce consequences for infants/families and hospital in following guideline e.g. length of stay, cost, reducing harm. **Feedback on behaviour**^**2**^: As per **Feedback** (Beliefs about consequences).
Social professional role and identity	**Social processes of encouragement, pressure, support**^**1**^: Clinical leads are respected clinicians who continually reinforce and role model guideline recommendations in practice.
Environmental context and resources	**Environmental changes**^**1**^: The Australasian Bronchiolitis guideline will be available in hard copy, electronic and via intranet to all clinicians. All teaching materials will be provided in hard and electronic copy. Clinical leads requested to incorporate guideline recommendations in staff induction sessions. **Prompts/cues**^**2**^: Clinical leads will be encouraged to use posters, screen savers, electronic prompts, email as reminders.
Skills	**Goal/target specified: behaviour or outcome**^**1**^: Hospitals will be encouraged to set improvement targets for guideline recommendations. **Monitoring**^**1**^: As per **Feedback** (Beliefs about consequences). **Increasing skills: problem solving, decision making, goal setting**^**1**^: Clinical leads will continually educate to increase knowledge, skills and confidence in diagnosing bronchiolitis, assessment and management. **Modelling/demonstration of behaviour of others**^**1**^**; Rehearsal of relevant skills**^**1**^**; Behavioural rehearsal/practice**^**2**^: Clinical leads will show clinicians a video of a clinician describing bronchiolitis to a family (for clinician teaching) and model this in clinical practice.
Social influences	**Social processes of encouragement, pressure, support**^**1**^**; Modelling demonstration of behaviour by others**^**1**^**; Social support or encouragement**^**2**^**; Modelling/demonstration of behaviour**^**2**^: Hospitals will receive a bronchiolitis information sheet for families/caregivers to support clinicians discussions with families. **Vicarious reinforcement**: As per **Vicarious reinforcement** (Beliefs about consequences)
Beliefs about capabilities	**Self-monitoring**^**1**^**; Increasing skills: problem solving, decision making, goal setting**^**1**^**; Rehearsal of relevant skills**^**1**^**; Social pressures of support, encouragement support**^**1**^ (as per **Social professional role and identity); Feedback**^**1**^ (as per **Beliefs about consequences**)**; Focus on past success**^**2**^**.** Clinical leads will provide encouragement on adherence to guideline recommendations using monthly audits to feedback on performance.

*TDF* Theoretical Domains Framework

*BCT* Behaviour Change Technique

^1^Michie et al. [4]

^2^Cane et al. [12]

As the five evidence-based recommendations being targeted are not independent, a pragmatic approach was taken where we assessed each of the domains and associated BCTs against all targeted behaviours we were aiming to influence. For example, the domain beliefs about consequences was identified for CXR, salbutamol and glucocorticoids. Using BCTs such as persuasive communication and feedback were deemed by the panel and research group as feasible and acceptable for all three targeted behaviours. Table 4 summarises the bronchiolitis intervention components developed including rationale.

**Table 4:** Overview of bronchiolitis interventions developed including rationale

Intervention and rationale	Content / techniques / additional information	Evidence source utilised in developing interventions	Influencing factors addressed, TDF domain, or factors taken into account by interventions
**1.Clinical leads** Rationale: Provide consistent credible, influential and trustworthy leadership. Increase knowledge and skills through education, influence and persuasion. Clinical leads ensure interdisciplinary and interdepartmental coverage.	Clinical leads (one nursing and one medical in both ED and inpatient paediatric units) for each hospital to both lead the study and train staff for duration of implementation period (May to November 2017). ‘Ideal characteristics’ of clinical leads discussed with hospitals.	EPOC review on local opinion leaders.	Power and influence within clinician groups rather than across. Clinician groups have their own systems to disseminate / implement changes. Leadership needs to be observable to keep momentum and give topic importance. Clinical leads for duration of study ensure consistency of education, role modelling, reinforcement of evidence-based practice and positivism. Given the intensity needed and to ensure maximum staff coverage, needed more than one clinical lead per area. Encourage communication and relationship building between ED and inpatient paediatric units: Bronchiolitis is a condition which spans the hospital journey, therefore collaboration between areas is important. Guide hospitals with their selection of appropriate clinical leads.
**2.Stakeholder Meeting** Rationale: Create site buy-in. Provide feedback on current bronchiolitis management. Knowledge of own practice variation is likely to drive change. Increase knowledge of intervention process. Identify and address any potential barriers.	Duration: 1 h Meeting with local stakeholders / clinical leads / clinical directors (nursing and medical). Provide information on study, expectations, attributes and importance of clinical leads. Provide opportunity to create buy-in at an organisational level and for senior leadership to express support. Start conversation with stakeholders (ED and inpatient paediatric units).	Hospital organisational factors.	Ensure all clinicians are aware of expectations of study involvement with aim to minimise chance of hospital or clinical leads dropping out over the duration of study. Opportunity for hospital clinicians to be together and create team cohesiveness from outset. Create buy-in from senior people involved in the implementation of the recommendations (organise top down, multi-disciplinary leadership).
	Present endorsed Australasian Bronchiolitis Guideline and discuss the 5 key guideline recommendations and evidence supporting these. Discuss international and local variation in bronchiolitis management.	The evidence-based Australian Bronchiolitis Guideline.	Discuss Australasian Bronchiolitis Guideline and recommendations, and international and local variation in practice. Strong evidence is pre-requisite for effecting change. Provide evidence-based recommendations using persuasive language.
	Review and discuss results of own hospital audit (20 ED and 20 inpatient bronchiolitis infants) and compliance to primary outcome (no CXR, salbutamol, glucocorticoids, antibiotics and adrenaline in first 24 h of presentation). Identify areas for improvement.	EPOC review on audit and feedback. Other documentation/information. Qualitative interview findings.	Acknowledging change is needed creates buy-in. Ensure the ‘key-people’ are aligned in their thinking. Create buy-in from clinical leads / stakeholders that change in practice is required with identification of the areas requiring most attention.
	Preliminary discussion of any anticipated local barriers and how to solve those.	Hospital organisational factors.	Intervention needs to fit in with local practices. Begin discussions between areas on how study and clinical leads will work in their hospital. Recognising and addressing any potential barriers at the beginning is more likely to optimise the hospital’s commitment and completion of the study.
**3.Train the Trainer Workshop** Rationale: Improve knowledge. Change beliefs. Optimise professional interdisciplinary and interdepartmental relationships. Motivate clinical leads as drivers of change.	One day event (8 h) – delivered in Melbourne. All four clinical leads invited to attend. Setting: off-site workshop venue. Delivered by senior research team clinicians / clinical opinion leaders.	EPOC review on local opinion leaders and continuing education meetings and workshops. Hospital organisational factors.	Clinical leads need to: - Have the clinical and leadership knowledge and skills in order to provide the local education / training / undertake requirements of study. - Understand the importance of their role. - Receive all interventions and resources required for local training. - Understand what is expected from them in terms of intervention delivery. - Have opportunity for time with other clinical leads from their hospital to plan intervention delivery and roles for the study.
	Set the scene: Australasian Bronchiolitis guideline, international / local variation in practice. Information on implementation science and implementation research.	Other documentation / information.	Set the scene / implementation capacity building. Gain buy-in on robust nature of how and why interventions have been developed and are to be delivered.
	Findings from qualitative study on barriers and facilitators to bronchiolitis management and intervention development. Rationale for intervention package. How to deliver intervention package. All study requirements. Planning time for clinical leads.	EPOC review on local opinion leaders. Qualitative interview findings. Intervention development.	Having knowledge of the process of intervention development will optimise buy-in. Clinical leads understanding of intended delivery method will ensure delivery of intervention with key messages relayed to their staff.
**4.Educational intervention delivery (PowerPoint)** Rationale: Improve knowledge. Increase skills. Change beliefs. Feedback on performance. Address barriers and enablers to evidence-based management. Reinforce importance of evidence-based management and consequences of not following recommendations. Positive reinforcement.	Education delivered by nursing and medical clinical leads to clinicians using PowerPoint presentation supplied (10–30 min). Additional slides provided giving more detail on evidence. Aim to train at least 80% of staff within one month and on-going training throughout implementation period. Bronchiolitis intervention package (detailed below). Role model to clinical leads what and how to teach their staff. Teach all participants together (nursing and medical).	EPOC review on local opinion leaders. Hospital organisational factors. Qualitative interview findings.	Designed with key messages and behaviour change techniques as detailed below. Role model delivery – emphasising persuasive and key messages. Management is both team-based, occurs across and between specialty teams as well as between medical and nursing. Potential staff availability issue, therefore clinical leads ideally to function as a team.
	Training materials addressing: 1. CXR Evidence re not performing CXR. Persuasive communication from credible sources / clinical leads. Reinforcement messages to follow guideline. Information on consequences of doing CXR. Role modelling of discussion with families about bronchiolitis and supportive care. Australasian Bronchiolitis guideline readily available. Fact sheets with more detailed evidence regarding CXR. Prompts. Posters. Audit and feedback.	Qualitative interviews.	TDF domains addressed: 1. Beliefs about consequences 2. Knowledge 3. Social influences 4. Skills
		Qualitative interviews. Hospital organisational factors.	TDF domain addressed: 1. Environmental context and resources
	2. Salbutamol Evidence re not using salbutamol. Persuasive communication from credible sources / clinical leads. Reinforcement messages to follow guideline. Information on consequences of giving salbutamol. Role modelling of discussion with families about bronchiolitis and supportive care. Australasian Bronchiolitis guideline readily available. Fact sheets with more detailed evidence regarding salbutamol. Prompts. Posters. Audit and feedback.	Qualitative interviews.	TDF domains addressed: 1.Beliefs about consequences 2. Knowledge 3. Social professional role and identity 4. Social influences
	3. Antibiotics Evidence re not using antibiotics. Persuasive communication from credible sources / clinical leads. Reinforcement messages to follow guideline. Information on consequences of giving antibiotics. Antibiotic stewardship. Role modelling of discussion with families about bronchiolitis and supportive care. Australasian Bronchiolitis guideline readily available. Fact sheets with more detailed evidence regarding antibiotics. Prompts. Posters. Audit and feedback.	Qualitative interviews.	TDF domains addressed: 1.Beliefs about consequences 2. Social influences 3. Knowledge
	4. Glucocorticoids Evidence re not using glucocorticoids. Persuasive communication from credible sources / clinical leads. Reinforcement messages to follow guideline. Role modelling of discussion with families about bronchiolitis and supportive care. Australasian Bronchiolitis guideline readily available. Prompts. Posters. Audit and feedback.	Qualitative interviews.	TDF domains addressed: 1.Beliefs about consequences 2. Knowledge 3. Social influences 4. Beliefs about capabilities
**5.Additional educational tools and materials** Rationale: Improve knowledge. Increase skill and confidence. Provide encouragement and support.	Clinician training video – role modelling how to talk with families about bronchiolitis (delivered by clinical leads to clinicians).	Qualitative interviews.	TDF domains addressed: 1. Knowledge 2. Skills 3. Social influences
	Fact sheets (delivered by clinical leads to clinicians).	Qualitative interviews. EPOC review on printed educational materials.	TDF domains addressed: 1. Knowledge 2. Social influences 3. Social professional role and identity 4. Beliefs about consequences
	Promotional materials – posters (placed in departments by clinical leads for clinicians and parents/caregivers).	Hospital organisational factors.	1. Knowledge 2. Environmental context and resources
	Parent/caregiver bronchiolitis information sheet (delivered by clinical leads to clinicians for use with parents/caregivers).	Qualitative interviews. Hospital organisational factors.	TDF domain addressed: 1. Knowledge 2. Social influences
**6.Audit and feedback** Rationale: Provide real-time feedback on targeted behaviours. Motivate by benchmarking. Promote goal / target specific action planning to optimise on-going improvement. Increase knowledge. Change beliefs.	Monthly audit and feedback cycles (7 months). Reports provided tabulated and graphical displays of hospitals performance compared to previous audits. Benchmark against top site. Disseminated regularly by clinical leads to clinicians using written, verbal methods of feedback.	EPOC review on audit and feedback. Sites provide monthly data.	TDF domain addressed: 1. Knowledge 2. Social professional role and identity
	Action planning in response to audit results (by clinical leads to clinicians).	Sites provide monthly data.	Action planning may improve practice – written and verbal. Clinical leads can target one behaviour at a time; use case review of non-compliant infant to discuss recommendations.

EPOC Effective Practice and Organisation of Care

*ED* Emergency Department

*TDF* Theoretical Domains Framework

*CXR* Chest X-ray

#### Identify evidence from systematic reviews of effects of interventions to inform the selection of intervention components

Findings from Cochrane EPOC reviews that focused on the effectiveness of interventions to influence the identified behaviours in the acute care setting were considered. **Additional** Table 1 includes the key findings from the reviews and the intervention components considered by the research team.

#### Identify feasibility, local relevance and acceptability of the intervention

The feasibility of delivering each of the proposed bronchiolitis interventions was discussed with the clinician panel and research group members e.g. delivering an education presentation to all staff in departments that have regular rotations of new staff; monthly audit and feedback cycles. With this in mind, we used a real-world approach using the group’s knowledge of the acute clinical demands, organisational context and constraints in order to make decisions on the feasibility and acceptability of the interventions.

Discussions resulted in the agreement on six bronchiolitis interventions: (1) Local stakeholder meetings, (2) Nomination of clinical leads (four in total - one medical and one nursing from both ED and paediatric inpatient units), (3) Train-the-trainer workshop (for all four clinical leads to attend), (4) Local educational materials targeting specific influencing factors, with delivery facilitated by clinical leads, (5) Promotional and other educational materials, and (6) Audit and feedback (Table 4).

## Discussion

This paper illustrates the stepped, theory and evidence informed process undertaken to develop targeted interventions aiming to improve the management of infants with bronchiolitis. The effectiveness of the six interventions developed has been robustly assessed via a multi-centre cRCT [26]. In this trial of 26 hospitals during the 2017 bronchiolitis season (May to November), with data from 3727 infants, the interventions were shown to improve bronchiolitis management by 14.1% (95% CI 6.5 to 21.7%) in hospitals randomised to the interventions compared to control hospitals who undertook usual dissemination practices of the Australasian Bronchiolitis Guideline [26]. This absolute change in care of infants with bronchiolitis is at the upper end of improvements shown in implementation cRCTs [38] and EPOC systematic reviews focusing on the effectiveness of interventions, predominantly to implement care, across healthcare settings [31–36] (Additional Table 1).

Using a systematic theory-driven approach during intervention development by targeting interventions to identified factors and determinants of practice, is more likely to increase intervention effectiveness than instinctively developing an intervention [3, 4, 39]. This stepped process has been used successfully in adult acute care settings aiming to improve the management of stroke [29] and minor traumatic brain injury [28] with interventions being assessed in cRCTs [10, 11]. While these approaches have been used to implement evidence-based practice, there are few frameworks to guide de-implementation with no ‘magic bullet’ or ideal intervention, despite the fact that de-implementation possibly presents a harder task than implementation [22]. To our knowledge this is the first time a structured theory-driven approach has been used to successfully explore barriers and enablers in the evidence-based management of bronchiolitis in acute care settings, then use BCTs to develop intervention components aiming to improve management and reduce low-value care. The Choosing Wisely De-Implementation Framework (CWDF) has recently been described, building on previous implementation science work [22]. Our stepped design successfully incorporated the first three phases described in the CWDF: Phase 0, identification of potential areas of low-value healthcare; Phase 1, identification of local priorities for implementation recommendations; and Phase 2, identification of barriers to implementing recommendations and potential interventions to overcome these. Phase 3, rigorous evaluation of the intervention, has been subsequently undertaken with robust evaluation of our interventions in a cRCT [26]. Phase 4 involves broad dissemination to all similar clinical settings. As with interventions to improve care, we theorise that the stepped process undertaken in developing our de-implementation interventions is more likely to change practice than if interventions were developed by chance or consensus opinion from experts.

A systematic review of the effectiveness of quality improvement strategies to improve inpatient bronchiolitis management demonstrated a reduction in unnecessary care in 14 trials identified [40]. While none were RCTs, and thus rated moderate quality of evidence at best, a variety of quality improvement interventions were effective for four of our low-value treatments targeted (CXR, salbutamol, antibiotics, and glucocorticoids). Unfortunately, no recommendation was given on any intervention being more effective, due to variability in study reporting. A systematic review of practice change interventions in paediatric emergency medicine highlighted lack of reporting of methodology being a barrier to future improvement efforts [41]. Other studies report interventions to reduce unnecessary care being developed by expert clinicians [42, 43]. While these interventions may have by chance addressed factors influencing the management of bronchiolitis they were not developed in a theory-informed manner. Detailed description of a theory-informed approach, clear rationale for intervention design, and explicit description of interventions that we describe are important for future replication as well as scaling up of effective interventions.

Our interventions were designed to target behaviours most likely to lead to non-evidence-based bronchiolitis management, addressing the majority of the identified TDF domains. The environmental context and resources domain posed challenges, as addressing time pressures within ED and acute care settings or changing the physical environment was beyond the scope of any pragmatic intervention. We addressed these challenges through provision of promotional and reminder materials and making the guideline available in hard and electronic copies. Interventions being feasible, practical and acceptable in the ED and paediatric inpatients units was considered important. Strategies to address these points included nursing and medical clinical leads in both ED and paediatric inpatient units, brief educational materials, and audits with succinct, timely and meaningful feedback. These real-world considerations increased the likelihood that interventions being acceptable within wider acute care environments.

Systematic reviews on intervention effectiveness in acute care settings are limited. Therefore, guidance was obtained from EPOC systematic reviews of intervention effectiveness across broad healthcare settings [31–36]. A recent systematic review of implementation strategies specific to child healthcare settings reported that single component interventions may be as, or more effective than multiple component interventions, with Computerised Decision Support (CDS) showing benefit [44]. While CDS is easily implemented within a single healthcare system, utilising a single CDS across multiple healthcare environments is problematic and not viable in our study. Educational interventions continue to be most commonly used for changing provider behaviour with positive results. Our educational intervention included important key messages, ensuring we targeted identified barriers and facilitators of the five non-evidence-based therapies.

The TDF was chosen as was the only framework available at the time that explicitly provided guidance on choosing intervention components. Subsequently, the Behaviour Change Wheel, linked to the TDF and a more simplified framework, has been developed with the central belief that capability, opportunity and motivation interact to produce behaviour [45]. Reviewing this guidance regarding the areas we were influencing, the BCTs and interventions we selected were comparable. Using this newer process would have resulted in similar interventions, suggesting that the interventions developed, and both frameworks are robust.

The major strength of our study is that a stepped theory-informed process was followed. The clinician interviews identified barriers and enablers to the evidence-based management of infants with bronchiolitis. Findings from the interviews ensured more informed understanding of the issues and challenges, from which BCTs were identified and operationalised in the interventions. Describing the stepped process ensures transparency and replicability of the method that may be applicable when developing interventions for other paediatric conditions or guideline implementation. The use of a panel and research group which included clinicians experienced in managing bronchiolitis from ED and paediatric inpatient units provided a comprehensive and complimentary skill base. This enabled decisions on the appropriateness of BCTs and intervention selection to be pragmatic and real world, while being evidence and theoretically based.

The final set of BCTs was generated by combining the five key evidence-based recommendations we were trying to influence. While this approach ensured no BCTs were left out, some BCTs were utilised across recommendations in order to preserve efficiency of the overall intervention package. Our panel and research group took into account that the recommendations were not independent, ensuring that interventions developed were feasible, practical and acceptable in the real-world of acute paediatric care.

Our interventions were targeted and contextualised to the Australian and New Zealand health care environment therefore applying them to other countries should be approached with caution. However, as variation in bronchiolitis management is an international problem, barriers and enablers we discovered and addressed may be similar to those found in other countries.

## Conclusion

Targeted interventions to improve the management of infants with bronchiolitis were developed using a stepped, evidence and theory-informed process. The TDF was used to: identify barriers and enablers to the evidence-based management of infants with bronchiolitis, identify BCTs most likely to influence these barriers and enablers, and select and develop appropriate interventions and methods of delivery. The intervention package has been evaluated in a large cRCT in Australia and New Zealand with results showing significant improvement in the management of infants with bronchiolitis. Thus, the development of theory and evidence informed interventions resulted in successful change in clinicians’ practice in the high patient throughput area of acute paediatrics. Future endeavours should assess the sustainability of this change.

## Supplementary Information

**Additional file 1: Table 1** Evidence from Cochrane EPOC reviews to inform bronchiolitis intervention components (adapted from Tavender et al 2015^1^). **Table 2** Barriers and enablers identified for bronchiolitis target behaviours by Theoretical Domains Framework.

## Data Availability

Not applicable.

## Abbreviations

BCT: Behaviour change technique
cRCT: Cluster randomised controlled trial
CDS: Computerised decision support
CWDF: Choosing wisely de-implementation framework
CXR: Chest x-ray
ED: Emergency department
EPOC: Effective practice and organisation of care
GRADE: Grading of recommendations assessment, development and evaluation
HRC: Health research council
NHMRC: National health and medical research council
PREDICT: Paediatric research in emergency departments international collaborative
RCT: Randomised controlled trial
TDF: Theoretical domains framework
TIDieR: Template for intervention description and replication
WIDER: Workgroup for intervention development and evaluation research
