# Supplementary material for: Development of targeted, theory-informed interventions to improve bronchiolitis management
Source: BMC Health Serv Res. 2021 Aug 3;21:769. doi: 10.1186/s12913-021-06724-6 (PMC8335893; doi:10.1186/s12913-021-06724-6)
Supplement: Supplementary file 1 — Additional file 1: Table 1 Evidence from Cochrane EPOC reviews to inform bronchiolitis intervention components (adapted from Tavender et al 20151). Table 2 Barriers and enablers identified for bronchiolitis target behaviours by Theoretical Domains Framework. [file 12913_2021_6724_MOESM1_ESM.docx]

**Additional Table 1** Evidence from Cochrane EPOC reviews to inform bronchiolitis intervention components (adapted from Tavender *et al* 2015^1^)

| **Cochrane review topic** | **Definition** | **Mechanism of action and practicality** | **Key findings** | **Effect sizes** | **Proposed implications for intervention components** |
| --- | --- | --- | --- | --- | --- |
| Continuing education meetings and workshops^2^ | Participation of healthcare providers in conferences, lectures, workshops or traineeships. | Didactic meetings target knowledge at the individual healthcare professional/peer group level. Interactive workshops target knowledge, attitudes and skills. Practicalities: commonly used with the main cost related to the release time for healthcare professionals and feasible in most settings. | Educational meetings alone or combined with other interventions can improve professional practice and the patient healthcare outcomes. The effect on professional practice tended to be small and varied between studies, and the effect on patient outcomes was generally less. It is not possible to explain the observed differences in effect with confidence but it appeared that higher attendance at the meetings was associated with greater effects, that mixed interactive and didactic education was more effective than either alone, and that the effects were less for more complex behaviours and less serious outcomes. | 81 randomised controlled trials (11,000+ health professionals). Median absolute improvement in care of 6.0 % (IQR +1.8 % to +15.3 %). | Mixed interactive education sessions and didactic education.  [Note: may have increased effect as clinicians generally do not want to prescribe or order potentially harmful or unnecessary medications or investigations]. |
| Local opinion leaders ^3^ | Use of individuals perceived by their colleagues as likeable, trustworthy and influential, who can educate and persuade clinicians to use best available evidence. | Target: knowledge, attitudes and social norms of their peer group. Dependent on the existence of intact social networks within professional communities. Practicalities: resources required include the process of the identification method, training of opinion leaders and potential additional service costs. | Opinion leaders alone or in combination with other interventions can be effective in promoting evidence-based practice, but effectiveness varies both within and between studies. The costs and cost effectiveness are unknown. These results are based on heterogeneous studies differing in terms of type of intervention, setting, and outcomes measured. In most studies, the role and actions of the opinion leader was not clearly described, and it is therefore not possible to say what the best way is to optimise their effectiveness. It is unclear whether methods to select opinion leaders is important or if effect differs if education is delivered by a single or multidisciplinary opinion leader teams. | 24 randomised controlled trials (337 hospitals and 350 primary care practices, 3,005 healthcare professionals, and 29,167 patients). Median absolute improvement in care of 10.8 % (IQR +3.5 % to 14.6 %). | Four local opinion leaders (clinical leads) to be selected at each hospital (two from emergency department (ED) and two from paediatric inpatients) with ideal characteristics and role being clearly described.  Clinical leads for the duration of the implementation period.  Importance of having nursing and medical clinical leads from ED and paediatric inpatients as infants with bronchiolitis are managed in both these areas.  Importance of nursing clinical leads to be articulated, as bronchiolitis management is supportive and largely nurse led. |
| Printed educational materials^4^ | Distribution of published or printed recommendations for clinical care including clinical practice guidelines, audio-visual materials and electronic publications. The materials may have been delivered personally or through mass mailings. | Target: knowledge and potential skill gaps of individual healthcare professionals. Can be used to target motivation when written as a ‘persuasive communication’ but little evidence of being used in this way. Practicalities: commonly used and relatively low cost and feasible in most settings. | Printed educational materials (PEMs) when used alone and compared to no intervention may have a small beneficial effect on professional practice outcomes. There is insufficient information to reliably estimate the effect of PEMs on patient outcomes, and clinical significance of the observed effect sizes is not known. The effectiveness of PEMs compared to other interventions, or of PEMs as part of a multifaceted intervention, is uncertain. | 14 randomised controlled trials and 31 interrupted time series studies. Median absolute risk difference in categorical practice outcomes was 0.02 when PEMs were compared to no intervention (range from 0 to +0.11). | Clinical guideline and key research publications to be provided.  Evidence fact sheets for not using antibiotics, salbutamol and performing a chest x-ray to be provided.  Bronchiolitis family information sheet to be provided.  Promotional material to be provided. |
| Audit and feedback^5^ | Any summary of clinical performance of healthcare over a specified period of time to change health professional behaviour as indexed by objectively measured professional practice in a healthcare setting or healthcare outcomes. | Target: ‘healthcare provider/peer groups’ perceptions of current performance levels and useful to create cognitive dissonance within healthcare professionals as a stimulus of behaviour change’. Practicalities: resources required to deliver audit and feedback including data extraction, analysis and dissemination costs. Feasibility dependent on availability of meaningful routine administrative data for feedback. | Audit and feedback generally lead to small but potentially important improvements in professional practice. The effectiveness of audit and feedback seems to depend on baseline performance and how the feedback is provided. Audit and feedback may be most effective when: (1) the health professionals are not performing well to start out with, (2) the person responsible for the audit and feedback is a supervisor or colleague, (3) it is provided more than once, (4) it is given both verbally and in writing and (5) it includes clear targets and an action plan. | 140 randomised controlled trials. Median adjusted RD was 4.3 % (IQR 0.5 % to 16 %). | Monthly audits to be undertaken with report produced detailing individual guideline recommendation and overall compliance.  Each site to be benchmarked (anonymously against top site).  Feedback to be provided by clinical leads in verbal and written format.  Clear targets and action plan to be encouraged.  [Note: May have more effect in sites that are low performing at beginning compared to high performing sites]. |
| On-screen point of care computer reminders^6^ | Patient or encounter specific information, provided verbally, on paper or on a computer screen, which is designed or intended to prompt a health professional to recall information. | Target: prompt health professionals to remember to do important things during patient interaction. Practicalities: resources necessary vary across the delivery mechanism. | Point of care computer reminders generally achieve small to modest improvements in provider behaviour. A minority of interventions showed larger effects, but no specific reminder or contextual features were significantly associated with effect magnitude. Further research must identify design features and contextual factors consistently associated with larger improvements in provider behaviour if computer reminders are to succeed on more than a trial and error basis. | 28 randomised controlled trials. Median absolute improvement of care (process adherence) was 4.2 % (IQR +0.8 % to +18.8 %). | Encourage the use of point of care reminders, ideally computer reminders but if not feasible paper reminders such as stickers on patient notes.  [Note: May not be feasible as some hospitals have no control over desktop screen savers and ability to instigate electronic reminders will be limited]. |
| Educational outreach visits^7^ | Use of a trained person who meets with providers in their practice settings to give information with the intent of changing the providers’ practice. The information given may have included feedback on the performance of the provider(s). | Target: an individual’s knowledge and attitudes (predominately target prescribing behaviours). Practicalities: considerable resources including the costs of detailers and preparation of materials. | Educational outreach visits alone or when combined with other interventions have effects on prescribing that are relatively consistent and small, but potentially important. Their effects on other types of professional performance vary from small to modest improvements, and it is not possible from this review to explain that variation. | 69 randomised controlled trials involving 15,000 + health professionals. Median adjusted risk difference (RD) in compliance with desired practice was 5.6 % (IQR 3.0 % to 9.0 %). The adjusted RDs were highly consistent for prescribing (median 4.8 %, IQR 3.0 % to 6.5 % for 17 comparisons), but varied for other types of professional performance (median 6.0 %, IQR 3.6 % to 16.0 % for 17 comparisons). EOVs appeared to be slightly superior to audit and feedback | [Note: Although it was found that EOVs were effective with its use in improving prescribing practice was deemed the most consistent result, the considerable cost of including this component as an intervention in 24 hospitals across territories, states and countries, was a reason for not including it as an intervention component]. |

^1^Tavender *et al.* (28)

^2^Forsetlund *et al.* (31)

^3^Flodgren *et al.* (33)

^4^Giguere *et al.* (34)

^5^Ivers *et al.* (36)

^6^Shojania *et al.* (35)

^7^O'Brien *et al.* (32)

EPOC – Effective Practice and Organisation of Care

IQR – Interquartile Range

ED – Emergency Department

PEM – Printed Educational Material

RD – Risk Difference

EOV – Educational Outreach Visits

**Additional Table 2** Barriers and enablers identified for bronchiolitis target behaviours by Theoretical Domains Framework

| **Target behaviour: CXR** | |
| --- | --- |
| **TDF Domains** | **Barriers and enablers** |
| Beliefs about consequences | Aware of risks of radiation exposure but believing that only a small amount of radiation is involved.  Doctors concern that the level of respiratory distress or deterioration may be that they are missing an alternative and treatable diagnosis.  Believing that performing CXR will confirm the diagnosis. |
| Knowledge | Lack of knowledge and experience in caring for infants with bronchiolitis. |
| Social influences | Doctors sensing parental pressure that a CXR will be performed.  Doctors getting pressure from other clinicians. |
| Environmental context and resources | Reduced senior medical support after hours.  Time pressures in ED to make decisions.  Regional hospitals:  Having less paediatric trained/experienced staff and higher numbers of overseas trained doctors who may practice differently.  Significant distance and time to tertiary care.  The requirement of a CXR prior to retrieval or transporting to tertiary care. |
| Skills | Lack of confidence and competence in diagnosing bronchiolitis. |

| **Target behaviour: Salbutamol** | |
| --- | --- |
| **TDF domains** | **Barriers and enablers** |
| Beliefs about consequences | Belief that some infants will get benefit from salbutamol and may improve enough to be able to be discharged, with others saying that there is no benefit in trialling salbutamol.  Belief that trialling salbutamol will do no harm.  Nurses and doctors indicating that a new guideline would be beneficial in changing beliefs, with some senior doctors suggesting that their practice wouldn’t change with new guideline. |
| Knowledge | Senior and junior clinicians’ lack of knowledge in regard to current evidence. |
| Social professional roles | Nurses supporting junior doctors in the management of infants with bronchiolitis.  Nurses assessment findings not being regarded when salbutamol use being questioned. |
| Social influences | Sense of parental and clinician pressure to trial salbutamol. |

| **Target behaviour: Antibiotics** | |
| --- | --- |
| **TDF domains** | **Barriers and enablers** |
| Beliefs about consequences | General belief that there is no benefit in prescribing antibiotics.  Mixed opinions on risks of over prescribing.  Confidence in advising antibiotics to be stopped.  Belief that antibiotics may be considered more in deprived populations due to high bronchiectasis population. |
| Knowledge | Lack of knowledge and experience in caring for infants with bronchiolitis. |
| Social influences | Doctors sensing parental pressure to prescribe antibiotics. |

| **Target behaviour: Glucocorticoids** | |
| --- | --- |
| **TDF domains** | **Barriers and enablers** |
| Beliefs about consequences | Clinician concern at potential harmful effects of glucocorticoids.  Confidence in advising glucocorticoids to be stopped. |
| Knowledge | Clinicians’ lack of knowledge in regard to current evidence. |
| Social influences | Doctors sensing parental pressure to prescribe glucocorticoids. |
| Beliefs about capabilities | Importance of maintaining good relationships with primary care providers. |

| **Target behaviour: Adrenaline** | |
| --- | --- |
| **TDF domains** | **Barriers and enablers** |
|  | Nil identified. Adrenaline is rarely used in Australia and New Zealand, with the exception of peri-arrest scenario in which its use is justified. |

CXR – Chest X-ray

TDF – Theoretical Domains Framework

ED – Emergency Department
